# Longitudinal Evaluation of Serum MOG-IgG and AQP4-IgG Antibodies in NMOSD by a Semiquantitative Ratiometric Method

**DOI:** 10.3389/fneur.2021.633115

**Published:** 2021-03-08

**Authors:** Luca Bollo, Pietro Iaffaldano, Maddalena Ruggieri, Claudia Palazzo, Mariangela Mastrapasqua, Alessia Manni, Damiano Paolicelli, Antonio Frigeri, Maria Trojano

**Affiliations:** Department of Basic Medical Sciences, Neurosciences and Sense Organs, University of Bari “Aldo Moro”, Bari, Italy

**Keywords:** NMO antibodies, NMOSD, NMO spectrum disorder, MOG (myelin oligodendrocyte glyco protein), neuroinflammation

## Abstract

**Background and purpose:** Immunoadsorption (IA) is an antibody-depleting therapy used to treat neuromyelitis optica spectrum disorder (NMOSD) associated to antiaquaporin 4 (anti-AQP4-IgG) and antimyelin oligodendrocyte glycoprotein (anti-MOG-IgG) serum autoantibodies. Our aim was to evaluate longitudinal changes of serum MOG-IgG and AQP4-IgG antibody titer and to correlate it with the clinical status.

**Methods:** Autoantibody titer and clinical features of two MOG-IgG+/AQP4-IgG– and two AQP4-IgG+/MOG-IgG– patients with NMOSD were collected at baseline (T0), after 6 IA courses (T1), and then 2 weeks (T2) and 6 months after treatment (T3). A fluorescent ratiometric assay was used for a quantitative detection of MOG and AQP4 antibodies, based on HEK-293 cells transfected with the full-length hMOG fused to GFP or h-AQP4-M23 isoform fused to m-cherry, respectively. We defined the antibody titer as MOG quantitative ratio (MOGqr) and AQP4 quantitative ratio (AQP4qr).

**Results:** In Case 1, the MOGqr dropped from 0.98 at T0 to 0.14 at T3, and in Case 2, it decreased from 0.96 at T0 to undetectable at T3. In Case3, the AQP4qr remained high: 0.90 at T0 and 0.92 at T3. In Case 4, the AQP4qr decreased from 0.50 at T0 to undetectable at T3. Complete recovery was found in Cases 1, 2, and 4.

**Conclusions:** Semiquantitative ratiometric method accurately detects even slight variation of MOG-IgG and AQP4-IgG titer, suggesting it may be useful to monitor the antibody titer during the disease course and maintenance immunotherapy.

## Introduction

Neuromyelitis optica spectrum disorders (NMOSD) are autoantibody-mediated chronic inflammatory diseases (1). Serum immunoglobulin G (IgG) autoantibodies binding to astrocytic aquaporin 4 (AQP4), the principal water channel of the central nervous system (CNS) (2), cause autoimmune astrocytopathies, characterized by recurrent attacks of the optic nerve, brainstem, and spinal cord (1). In some patients, presenting with NMOSD clinical and radiological features, serum IgG autoantibodies against myelin oligodendrocyte glycoprotein (MOG) are detected (3). MOG-IgG autoantibodies (Abs) are associated with a wide spectrum of demyelinating central nervous system (CNS) disorders, such as optic neuritis (ON), myelitis, clinically isolated syndrome (CIS), NMOSD, acute demyelinating encephalomyelitis (ADEM), or multiphasic demyelinating encephalomyelitis (MDEM) (4). These clinical syndromes are now frequently referred as MOG encephalomyelitis (MOG-EM) or MOG antibodies disease (MOGAD) (5). According to the revised NMOSD diagnostic criteria, patients without evidence of AQP4-IgG can be assigned to NMOSD, even though their relevance as nosological entity is currently under discussion (6).

Magnetic resonance imaging (MRI) is one of the most important diagnostic tools to distinguish multiple sclerosis (MS) from AQP4-positive NMOSD and MOGAD. The Wingerchuck 2015 criteria have shown 90.9% sensitivity and 89.7% specificity in identifying all types of NMOSD (7).

The gold standard test for detecting MOG-IgG and AQP4-IgG is the cell-based assay (CBA) that, compared to enzyme-linked immunosorbent assays (ELISA) or immunoprecipitation assays, shows greater sensitivity and specificity (8, 9). The sensitivity of CBA ranges between 80 and 100%, while specificity ranges between 86 and 100% (3).

In NMOSD patients, the treatment of acute attacks is usually represented by 1,000 mg intravenous methylprednisolone (IVMP) for 3–5 days (8), although a treatment escalation to 2,000 mg IVMP may be administered in case of poor response (10). However, IVMP in NMOSD patients led to complete recovery just in 19–35% of myelitis and in 33–53% of acute attack of ON (8); therefore, more aggressive treatments of attacks have been suggested. In particular, a study by Kleiter et al. (10) suggested that the first-line therapy with apheresis may be superior to IVMP in attacks involving the spinal cord. Apheresis therapy aims to eliminate antibodies and other proinflammatory factors from patients' sera. Two main techniques are used: immunoadsorption (IA) and plasma exchange (PLEX). In both procedures, the plasma is separated from the whole blood including albumin, complement factors, and immunoglobulins. In IA, the plasma needs to pass through an IA device before reinfusion. In PLEX, the filtered plasma is rejected, and either fresh-frozen plasma or 5% albumin solution is added before being instilled. No superiority was shown for one of the two apheresis techniques (11).

We report a clinical case series of four NMOSD patients who came to our center with an acute relapse, treated with IA as second line therapy, after no recovery with IVMP.

## Materials and Methods

Two NMOSD patients MOG-IgG+/AQP4– and two NMOSD patients AQP4-IgG+/ MOG-IgG– were examined. At baseline, all patients underwent brain and spinal cord MRI, lumbar puncture (LP), and infectious diseases [*Borrelia burgdorferi, Treponema pallidum, Toxoplasma*, Herpes virus types 1 and 2, varicella zoster, *Cytomegalovirus*, Epstein–Barr virus, hepatitis A, B, and C virus, and human immunodeficiency virus (HIV)] and autoimmunity tests [antinuclear antibodies (ANA), antineutrophil cytoplasmic antibody (ANCA), cardiolipin antibodies or phospholipid/glycoprotein beta-2-antibodies, rheumatoid factor]. All patients were treated with IVMP (5–13 × 1000 mg) as first-line therapy with no recovery and then with IA using tryptophan (TR350) as second-line therapy (median number of IA courses = 10; range, 7–13).

Clinical status and serum MOG-IgG and AQP4-IgG Abs were assessed at admission before IA therapy (T0), after six IA courses (T1), and then 2 weeks (T2) and 6 months after treatment ends (T3) (Tables 1, 2).

**Table 1:** MOGqr and visual acuity (VA) in case 1 and 2 collected over time.

	**T0**		**T1**		**T2**		**T3**	
	**MOGqr**	**VA**	**MOGqr**	**VA**	**MOGqr**	**VA**	**MOGqr**	**VA**
Case 1	0.98	5/10	0.80	7/10	0.5	10/10	0.14	10/10
Case 2	0.96	<0.1	0.25	3/10	<0.02	3/10	<0.02	3/10

*MOGqr, MOG quantitative ratio; VA, visual acuity; T0, onset, T1, after 6 IA courses, T2, two weeks after IA treatment, T3, 6 ± 2 months after IA therapy*.

**Table 2:** AQPqr and EDSS in case 3 and 4 collected over time.

	**T0**		**T1**		**T2**		**T3**	
	**AQP4qr**	**EDSS**	**AQPqr**	**EDSS**	**AQPqr**	**EDSS**	**AQPqr**	**EDSS**
Case 3	0.90	7.5	0.65	7.0	0.70	6.5	0.92	8.0
Case 4	0.50	3.5	0.60	3.0	<0.02	2.0	<0.02	2.0

*AQP4qr, AQP4 quantitative ratio; EDSS, Expanded Disability Status Scale; T0, onset; T1, after 6 IA courses; T2, two weeks after IA treatment; T3, 6 ± 2 months after IA therapy*.

Visual function testing was performed by standardized protocol under standardized conditions, and visual acuity (VA) was assessed with a retroilluminated Snellen letter chart (12).

### Cell Culture and Transfection

Serum MOG-IgG and AQP4-IgG were measured by an in-house CBA, the gold standard test according to the international recommendations for NMOSD (13) and MOGAD (6).

**Figure 1:**
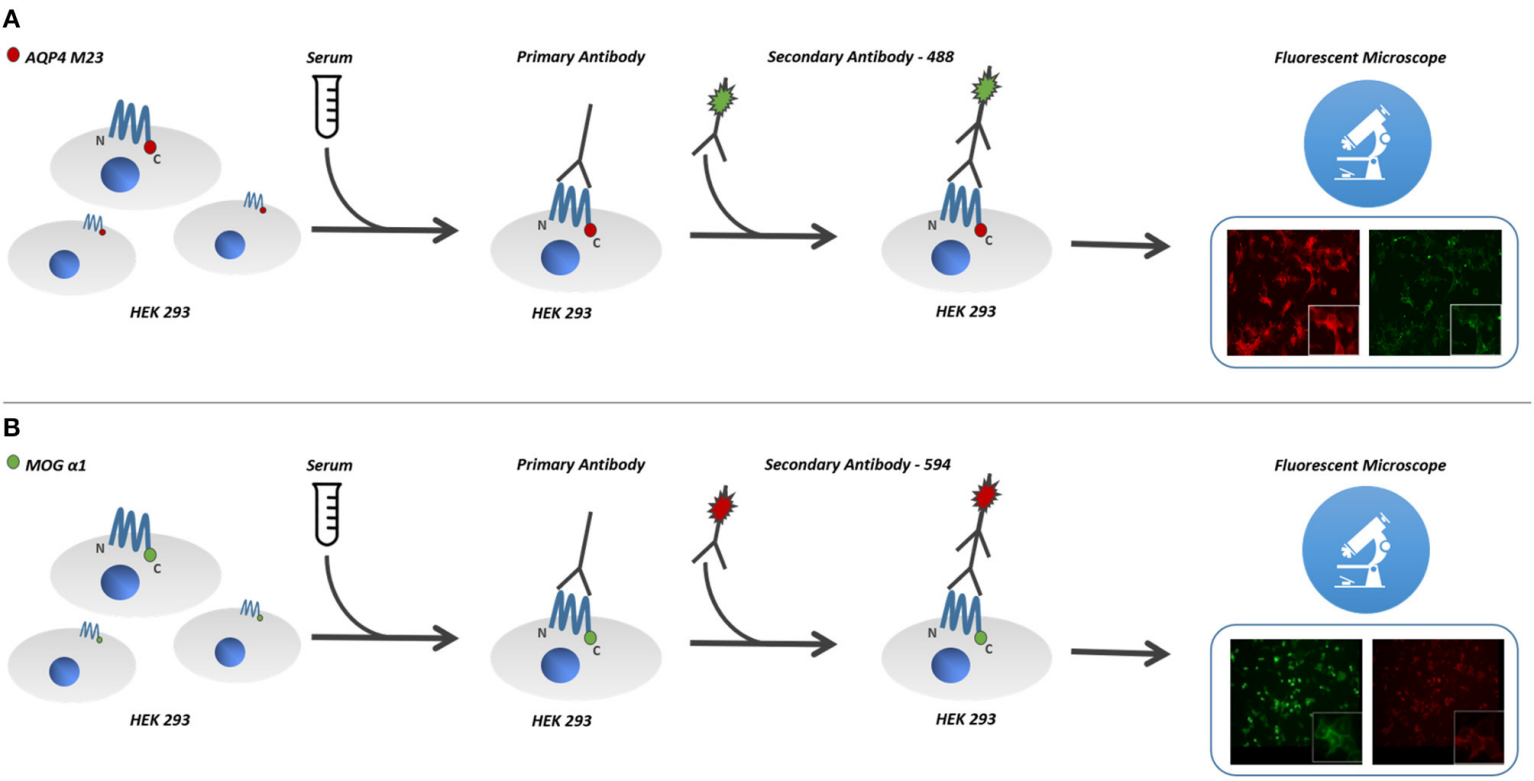
Cultured human cells (HEK293 cells) were transfected with the antigen, MOG or AQP4, and used as substrate in an indirect immunofluorescence assay. For MOG-IgG detection, cells were stably transfected with hMOG-α1 splice variant complementary DNA (cDNA) (FL-MOG-α1) cloned into pCMV6-AC-GFP plasmid. **(B)** Cells were stably transfected with hMOG construct or empty vector and used as the substrate for live cell-based assays (CBAs). **(A)** HEK-293 cells were also transfected with pmCherry-hAQP4-M23 expression vector, cloned to obtain M23mCherry stable expressing cell line. **(B)** Serum MOG-IgG was detected on the surface of MOG expressing cells, using goat antihuman 568 Alexa-Fluor-conjugated secondary antibodies, **(A)** while AQP4-IgG bound antibodies were detected using goat antihuman 488 Alexa-Fluor-conjugated secondary antibodies. AQP4-M23-IgG and MOG-IgG binding was determined using live-cell-staining immunofluorescence technique. MOG, myelin oligodendrocyte glycoprotein; AQP4, aquaporin-4 channel protein; HEK-293, human embryonic kidney 293 cells.

Cultured human cells (HEK293 cells) were transfected with the antigen, MOG or AQP4, and used as substrate in an indirect immunofluorescence assay (14) (Figures 1A,B).

The full-length hMOG-α1 splice variant complementary DNA (cDNA) (FL-MOG-α1) cloned into pCMV6-AC-GFP plasmid was kindly provided by Prof. Raffaele Iorio (Università Cattolica del Sacro Cuore, Rome, Italy). HEK-293 cells were grown in Dulbecco's high glucose medium with stable glutamine added with 10% fetal bovine serum and 100 U/ml penicillin/100 μg streptomycin (Invitrogen, Milan, Italy). Cells were stably transfected with hMOG construct or empty vector and used as the substrate for live cell-based assays (CBAs). Transfections were performed using Lipofectamine 2000 transfection reagent (Invitrogen, Milan, Italy) following the manufacturer's protocol (Figure 1B)

HEK-293 cells were also transfected with pmCherry-hAQP4-M23 expression vector, cloned as described elsewhere (15), to obtain M23mCherry stable expressing cell line. Stable clones were selected for 2 weeks in medium with 0.8 mg/ml geneticin and maintained thereafter in 0.4 mg/ml geneticin. Transductions resulted in almost 100% of MOG- or AQP4-M23-expressing cells (Figure 1A).

### Quantitative Analysis of Serum Antibodies Titer

AQP4-M23-IgG and MOG-IgG binding was determined using live-cell-staining immunofluorescence technique as previously described (15). Briefly, transfected cells cultured on glass coverslips were exposed to the sample sera (1:20 diluted) for 1 h at room temperature. Serum MOG-IgG was detected on the surface of MOG-expressing cells, using goat antihuman 568 Alexa-Fluor-conjugated secondary antibodies (Invitrogen, Life Technologies, Carlsbad, CA), while AQP4-IgG bound antibodies were detected using goat antihuman 488 Alexa-Fluor-conjugated secondary antibodies (Invitrogen Life Technologies, Carlsbad, CA). In order to evaluate unspecific background staining, we routinely performed serum antibody staining using empty vector transfected cells for both immunoassays. Coverslips containing cells where observed using a DMRXA fluorescence microscope (Leica Microsystems) provided with a DFC700T color camera.

The antibody titer was performed by a ratiometric method using the cells expressing the fluorescent protein (MOG-GFP, AQP4-mcherry). In detail, for each cell line, two microscopy fields were randomly chosen using a 20× objective. For each field, four different regions containing two to four cells each were analyzed. Fluorescent intensity of each region was measured with a grayscale, and background subtracted values were given as ratio of red/green for MOG antibody titer and green/red for AQP4 antibody titer. Values range from 0 for no staining to 1 for maximum antibody binding.

Therefore, we defined these values as MOG quantitative ratio (MOGqr) and AQP4 quantitative ratio (AQP4qr).

## Case Description and Results

Case 1 was a Caucasian 26-year-old woman with a 10-year history of migraine and a spontaneous abortion in 2016. In October 2018, 2 months after delivery, during breastfeeding, she developed acute bilateral blurred vision and retro-orbital pain on eyes movement and moderate frontal headache. Brain and orbital MRI demonstrated contrast enhancement in both optic nerves, compatible with bilateral optic neuritis, supratentorial nonspecific white matter lesions not meeting Paty and Barkhof criteria for MS (16), and a normal spinal MRI. The patient was admitted to our department after three cycles of IVMP (17 g total) leading to no recovery.

**Figure 2:**
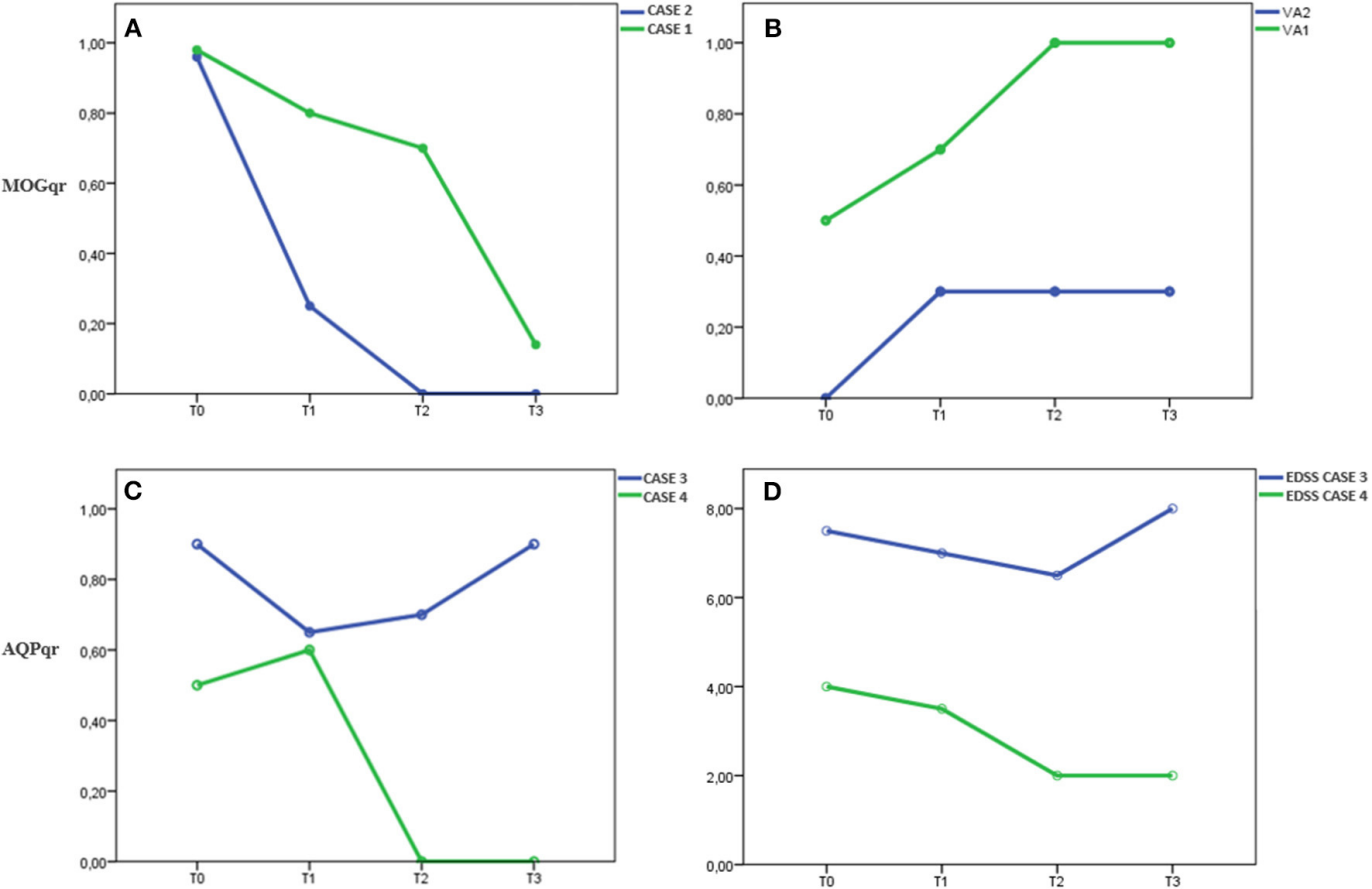
In Cases 1 and 2, **(A)** MOGqr decrease during time, **(B)** as the VA improve until complete recovery of starting VA in both patients. The titer remained low (Case 1) or undetectable (Case 2) during the IA treatment and at last FU (T3). In Case 4, **(C)** the AQPqr decrease during time with **(D)** clinical improvement of the patient. In Case 3, **(C)** the AQPqr was slightly reduced with almost no recovery at T2, and **(D)** at T3, the AQPqr increased and the clinical condition had worsened. MOGqr, MOG quantitative ratio; VA, visual acuity; AQP4qr, AQP4 quantitative ratio; EDSS, Expanded Disability Status Scale; T0, onset; T1, after 6 IA courses; T2, 2 weeks after IA treatment; T3, 6 ± 2 months after IA therapy.

Neurological examination was unremarkable except for bilateral visual loss, right visual acuity (RVA) 7/10, and left visual acuity (LVA) 5/10. Visual field examination revealed a bilateral large central scotoma more prominent in the left eye. Optical coherence tomography (OCT) examination evidenced a thinning of retinal nerve fiber layer (RNLF) in both eyes, greater in the left one. LP revealed mild blood–cerebrospinal fluid (CSF) barrier dysfunction and CSF-restricted IgG oligoclonal bands (OCB). MOG antibodies were positive with a MOGqr = 0.98 at T0. The patient was treated with 13 courses of IA; the MOGqr decreased to 0.8 at T1, with partial recovery of VA (RVA 10/10, LVA 7/10), and 0.7 at T2 with complete recovery of VA (Table 1). She was discharged with oral steroid for 3 months, and in June 2019, she started Azathioprine (AZA) 2 mg/kg (150 mg/day). Twelve months after the IA treatment (T3), the MOGqr was 0.14, and a normal VA was documented (Figures 2A,B).

Case 2 was a 26-year-old man, Caucasian, with a history of congenital rubella syndrome expressed by eye abnormalities (retinitis and cataract) and consequent visual impairment (RVA 5/10 and LVA 3/10). In 2014, he had an unprovoked epileptic seizure; the brain MRI scan showed multiple T2-hyperintense lesions in the white matter that were attributed to the previous rubella infection; and no contrast-enhancing lesions were found. No antiepileptic drug was started. In November 2018, he developed a bilateral optic neuritis with a worsening of visual acuity (RVA 3/10, functional blindness in the left eye). CSF examination revealed mild pleocytosis (7 leukocytes/μl), blood–CSF barrier dysfunction, and CSF-restricted OCB. Brain MRI examination showed optic nerves' swelling with Gd-enhancement and several T2-hyperintense lesions involving the subcortical and supratentorial white matter, sparing the corpus callosum. Spinal MRI was normal. MOG-IgG antibodies were detected with MOGqr = 0.96 at T0. The patient was treated with 13 cycles of IA; the MOGqr decreased to 0.25 at T1 with a slight improvement of VA (Table 1). At T2, the MOGqr was under the mean healthy control values with a complete recovery of VA, and the patient started a therapy with rituximab (RTX) 2 × 1,000 mg, 2 weeks apart. At T3, the VA was unmodified, and the MOG-IgG antibodies were still undetectable in the serum (Figures 2A,B).

Case 3 was a 39-year-old Caucasian woman. In 2008, she was hospitalized for an episode of cerebral salt wasting syndrome (CSWS) (17). The brain MRI scan revealed a T2-hyperintense diencephalic lesion without Gd enhancement, and the spinal MRI was normal. CSF revealed normal cell count, moderate blood–CSF barrier dysfunction, and negative OCB. No diagnosis of NMOSD was made, and no disease-modifying drugs (DMDs) were started. In 2019, she developed bilateral dysesthesia and four limbs palsy, with severe bladder dysfunction [Expanded Disability Status Scale (EDSS) 7.5]. MRI showed two longitudinally extensive transverse myelitis (LETM) T2 lesions extending from C2 to C7 and from T3 to L1, both with Gd enhancement. AQP4 antibodies were positive (AQPqr = 0.90) at T0. The patient achieved a partial recovery (EDSS 7.0) at T1 with a slight decreasing in AQPqr = 0.65. At T2, the clinical status minimally improved (EDSS 6.5), while the AQP-IgG titer increased (AQPqr = 0.7) (Table 2).

The patient started RTX (2 × 1,000 mg, 2 weeks apart), but at T3, she developed a relapse with an acute myelitis with severe paresis of the four limbs (EDSS 8.0); she was tested for AQP-IgG, and the titer rose up again (AQPqr = 0.92). The spinal MRI showed a new T2-hyperintense lesion with Gd enhancement from C7 to T1 (Figures 2C,D).

Case 4 was a 44-year-old woman, with a medical history characterized by several autoimmune diseases (Vitiligo, Hashimoto's thyroiditis, autoimmune proctitis). In July 2018, she developed an area postrema syndrome (APS) and moderate lower limbs palsy with a partial spontaneous regression in 1 week (EDSS 2.0). In October 2018, she complained of increasing dysesthesia in lower limbs and impaired ambulation. Neurological examination revealed mild lower-limb paresis and hypoesthesia with urinary incontinence (EDSS 3.5). The spinal cord MRI showed a T2-hyperintense lesion, without Gd enhancement from the obex to C5 and an LETM with Gd enhancement in the thoracic cord. CSF showed normal cell count and slightly elevated protein levels but no intrathecal IgG synthesis (no OCB, QIgG normal). She was tested for AQP4-IgG antibodies, and the AQPqr was 0.5 at T0. The treatment with IA (13 courses) was started. At T1, the AQPqr was 0.6. After the IA treatment (T2), the AQP4qr was undetectable in serum (Table 2); the neurological examination of the patient revealed very mild foot flexor and extensor paresis bilaterally (4/5), with no sensory symptoms and an EDSS score of 2; the patient started RTX (2 × 1,000 mg, 2 weeks apart). At T3, the neurological examination was stable (EDSS 2.0), the MRI did not show new brain or spinal cord lesions, and the AQP4-IgG was still under the mean negative control value (Figures 2C,D).

## Discussion and Conclusions

Four NMOSD patients came to our attention for an acute relapse without recovery after IVMP. They were treated with IA therapy as second-line therapy and 52 IA single procedures. We evaluated the clinical examination and the MOG-IgG and AQP4-IgG abs titer at different times of relapse treatment and at last follow-up with a semiquantitative ratiometric method.

Live CBA represents the “gold standard” test for serum MOG-IgG and AQP4-IgG detection. This methodology has allowed to identify these antibodies in non-MS acquired demyelinating CNS syndromes (18). However, this technique requires the microscopy analysis done by at least two operators who give a qualitative score, according to the intensity of the staining, and it is time consuming, since it requires serum dilution steps.

In our study, a ratiometric method, based on a two-color detection, is used to give a more accurate MOG (red/green) and AQP4 antibody titers (green/red). The major strengths of this method are the following: (a) a less operator-dependent technique, (b) more reliable, (c) quicker to perform since serial dilutions are not required, and (d) capable to detect slight variation of the antibody titration.

Using this ratiometric method, it was possible to correlate the antibody titer to the clinical improvement after a specific therapy.

Cases 1 and 2 were two young adults with severe BON: case 1 with onset 2 months after delivery and case 2 with a possible onset, many years before the visual disturbance, with an unprovoked epileptic seizure. Indeed, there is growing recognition of seizures as a clinical manifestation of MOGAD (19). We correlated the MOGqr titers with the improvement of VA in the most affected eye, since they had no other neurological dysfunctions. They showed a progressive improvement of VA in both eyes until complete recovery that correlated with an antibody levels decrease (Figures 2A,B). At last follow-up (T3), they still presented low MOG-IgG titer, and no evidence of disease activity was observed.

As occurred in MOG-IgG-positive patients, in case 4, the level of AQP4-IgG decrease correlated with the clinical improvement, with a recovery to the starting clinical status (EDSS 2.0). At T3, the titer remained under the mean negative control value, and case 4 did not present clinical relapses and MRI disease activity.

In case 3, high values of AQP4qr were found at T1 and T2, and the patient had just a slight recovery as result of the aphaeretic treatment (T2: AQPqr = 0.70 and EDSS 6.5). At T3, she developed a new relapse despite the starting of DMD therapy with a further increase in AQP4qr to 0.90 and a clinical worsening (EDSS 8.0) (Figures 2C,D).

We dosed the MOG-IgG and AQP4-IgG by a semiquantitative ratiometric method, which is accurate to detect even slight variations of the antibody titer over time as shown in our patients. We observed a possible relationship between the drop of MOG-IgG and AQP4-IgG titer and the clinical improvement (Cases 1, 2, and 4), whereas in Case 3, the persistence of high levels of AQP4qr was associated with a slight recovery at T2 and a new relapse at T3 (Figure 2).

A previous study tried to investigate the prognostic role of the AQP4-IgG titer, dosed with an enzyme-linked immunosorbent assay (ELISA), and no significant differences were found in any clinical characteristic in NMOSD patients, by proposing that seropositive patients generally have the same clinical course regardless of antibody titer (20).

Nevertheless, relapsing clinical course was observed in a subset of patients with a persistent MOG-IgG-positive status compared to patients who did not experienced a clinical relapse after conversion to seronegativity under maintenance immunotherapy (21). The major limitation of our study is the small cohort of patients. However, our results may suggest that using a method more sensitive to slight variations in the antibody titer could lead to a more accurate definition of patients serostatus trend even under immunomodulant/immunosuppressant treatment.

Further studies, including a large cohort of patients with longitudinal titration and clinical follow-up, are warranted to confirm the role of antibody titer in monitoring disease activity and treatment response.

## Data Availability Statement

The raw data supporting the conclusions of this article will be made available by the authors, without undue reservation.

## Ethics Statement

Ethical review and approval was not required for the study on human participants in accordance with the local legislation and institutional requirements. The patients/participants provided their written informed consent to participate in this study. Written informed consent was obtained from the individual(s) for the publication of any potentially identifiable images or data included in this article.

## Author Contributions

LB, AF, PI and MT conceptualized the study, had full access to all data and take responsibility for the integrity of the data and the accuracy of the data analysis. LB, AF, PI and MT contributed to the data analysis and the writing of the manuscript. MR analyzed CSF data. CP and MM provided to analized the serum samples. All the authors contributed to the data interpretation and reviewed and approved the final version.

## Conflict of Interest

MT has served on scientific Advisory Boards for Biogen, Novartis, Roche, Merck, and Genzyme; has received speaker honoraria from Biogen Idec, Merck, Roche, Teva, Sanofi-Genzyme, and Novartis; and has received research grants for her Institution from Biogen Idec, Merck, Roche, and Novartis. PI has served on scientific advisory boards for Biogen Idec and has received funding for travel and/or speaker honoraria from Sanofi-Aventis, Biogen Idec, Teva, and Novartis. DP received advisory board membership, speaker's honoraria, travel support, research grants, consulting fees, or clinical trial support from Almirall, Bayer Schering, Biogen, Celgene, Excemed, Genzyme, Merck, Mylan, Novartis, Sanofi, Roche, and Teva. The remaining authors declare that the research was conducted in the absence of any commercial or financial relationships that could be construed as a potential conflict of interest.
